# Cross-Sectional Survey of Former International Emergency Medicine Fellows 2010–19

**DOI:** 10.5811/westjem.2020.7.45999

**Published:** 2020-10-06

**Authors:** Shama Patel, Alyssa Green, Michelle Feltes, Heike Geduld, Andrea G. Tenner

**Affiliations:** * Columbia University, Department of Emergency Medicine, New York City, New York; † Presbyterian Hospital System, Department of Emergency Medicine, Albuquerque, New Mexico; ‡ Stanford University, Department of Emergency Medicine, Palo Alto, California; § Stellenbosch University, Department of Emergency Medicine, Cape Town, South Africa; ¶ University of California, San Francisco, Department of Emergency Medicine, San Francisco, California; || University of Florida - Jacksonville, Department of Emergency Medicine, Jacksonville, Florida

## Abstract

**Introduction:**

International emergency medicine is a new subspecialty within emergency medicine. International emergency medicine (EM) fellowships have been in existence for more than 10 years, but data is limited on the experiences of the fellows. Our goal in this study was to understand the fellowship experience.

**Methods:**

The study employed a cross-sectional survey in which participants were asked about their demographics, fellowship program, and advanced degree. Participants consisted of former fellows who completed the fellowship between 2010–19. The survey consisted of both closed and open-ended questions to allow for further explanation of former fellows’ experience. Descriptive analysis was conducted on the quantitative survey data while content analysis was conducted to ascertain salient themes from the open-ended questions.

**Results:**

We contacted 71 former fellows, of whom 40 started and 36 completed surveys, for a 51% response rate (55.6% women). Two-year fellowships predominated, with 69.4% of respondents. Prior to fellowship, a subset of fellows spoke the native languages of their service sites: French, Spanish, Haitian Creole, Mandarin, or Kiswahili. Half the respondents spent 26–50% of their fellowship in field work, with 83.3% of institutions providing direct funding for this component. Many respondents stated a need for further institutional support (money or infrastructure) for fieldwork and mentoring. Non-governmental organizations comprised 29.7% of respondents’ work partners, while 28.6% were with academic institutions in country, focused mostly on education, health systems development, and research. The vast majority (92%) of respondents continued working in global EM, with the majority based in American academic institutions. Those who did not cited finances and lack of institutional support as main reasons.

**Conclusion:**

This study describes the fellow experience in international EM. The majority of fellows completed a two-year fellowship with 26–50% of their time spent in fieldwork with 83.3% of institutions providing funding. The challenges in pursuing a long-term career in global EM included the cost of international work, inadequate mentorship, and departmental funding.

## INTRODUCTION

Emergency medicine (EM) is a relatively new specialty with a variety of subspecialties, which have been growing in number and popularity. The international EM fellowship (IEMF) emerged over 10 years ago as a subspecialty providing public health training, experiences in resource-limited settings, and research and education in international health.^1^ IEMFs are aimed at EM trainees focused on emergency care provision and development in resource-limited settings such as low-and-middle-income countries (LMIC). While fellowship goals, objectives, and skills have been outlined previously,^1^–^4^ this information has not always been easily available to those applying.^5^

The fellowship attracts individuals interested in working with LMICs and in resource-constrained areas through direct service provision, as well as through research, EM education, health systems development, and humanitarian and disaster response. Over 20 academic institutions across the United States now offer IEMFs with projects throughout North and South America, Africa, the Middle East, and Asia. These fellowships are governed by the IEMF Consortium. Many offer an advanced degree in public health, global sciences, tropical medicine, or education. Each fellowship offers slightly different foci based on the goals of the fellowship and institution, faculty expertise, and existing country partnerships. The programs are not accredited by the Accreditation Council for Graduate Medical Education, and consequently there is a dearth of information on the fellowships themselves and experiences of the fellows. Now that IEMFs have graduated fellows for 10 years, this is an opportune time to describe the fellowship experience.

### Aims

Our goal was to describe and map the experiences of the IEMF fellows both domestically and abroad. We provide data that can be used to improve IEM training.

## METHODS

### Study Design and Population

We employed an electronic cross-sectional survey of all fellows who graduated from an IEMF at a US institution from 2010–2019. Current IEMFs were identified through the IEMF Consortium, which provided the fellowship directors’ email addresses. All current, active IEMFs are part of this consortium, but fellowships that have since closed or are inactive are not included. The consortium, in its role as oversight body for IEMFs, provided the most direct way of contacting fellows. Fellowship directors had the option to provide us with the emails of the former fellows or directly email the former fellows an anonymous link with consent to participate in the study. Institutional review board approval for the study was obtained by each author’s affiliated institution prior to study conduction.

**Population Health Research Capsule**

What do we already know about this issue?

*Information is limited on the experiences of international emergency medicine fellows (IEMF), whose focus is public health and who often become leaders in global health*.

What was the research question?

What motivates IEMFs to enter fellowship and what do they experience during fellowship and post-fellowship careers?

What was the major finding of the study?

*Most became IEMFs to professionalize their interest in global EM. Those who left the field cited finances*.

How does this improve population health?

*IEMFs work in global and population health after fellowship. Learning from their experiences can help create an even more effective cadre of professionals*.

### Survey Content and Administration

Survey participants were asked about their demographics, motivation for entering fellowship, fellowship program content and outcomes, advanced degree, if obtained, and post-fellowship activities. The survey consisted of a mix of closed and open-ended questions to allow for further elaboration. The survey was distributed using Qualtrics (Provo, UT) from April 29–May 15, 2019. Two additional follow-up emails were sent to the fellowship directors to ensure that as many former fellows as possible would be included in the study.

### Data Analysis

Quantitative data were collected via an anonymous online survey through the Qualtrics software. We conducted descriptive analyses on the data including geo-mapping of field sites. We analyzed qualitative data using content analysis. Themes were derived from the data by two independent, separate coders, and the derived themes were compared and agreed upon for the final analysis.

## RESULTS

### Demographics

Response rate was 51% (36/71). Respondents included slightly more women than men, with most between the ages of 35–44 (Table 1). Only 36.1 of respondents had an additional advanced degree (besides a medical or osteopathic degree) prior to starting the fellowship with the majority attending a two-year fellowship (69.4%). With regard to languages spoken, 43.8% reported the ability to speak another language besides English prior to starting the fellowship, which did not have a significant impact on where their fieldwork was conducted.

### Fellowship Demographics Results

Most respondents went to a two-year program (69.4%) earning a master of public health degree (69.5%) during fellowship (Table 2).

### Motivations and Perceptions of Training

Fellows reported that they decided to enter the fellowship to develop a humanitarian aid career, enter academic international EM, have dedicated field time, develop research skills, and obtain mentorship. One respondent stated he wanted to enter an IEMF to “*professionalize [his] interest in global health.”* Respondents elaborated and stated the most valuable components of the degree were learning public health methodology (specifically epidemiology, biostatistics, population health, monitoring, and evaluation), becoming subject matter experts, and having the opportunity to network during fellowship. The least valuable components commonly reported were limited statistics and classes aimed at non-clinicians.

Respondents worked an average of 719 hours per year clinically (interquartile range of 161.5 hours) at the fellowship institution with 88.8% having a faculty appointment during fellowship; 88.9% of respondents’ fellowships had existing field sites with over 80.6% working at those sites. Respondents stated that institutional support was in the form of pre-existing fieldwork/sites, funding for travel, clinical scheduling flexibility, and research support. Respondents stated that further institutional support could be provided through *“more autonomy and reduction of barriers to fieldwork”;* research and scholarly mentorship; *“more cross-institution mentorship on how to prepare for a further career in international EM;, more mentoring for early faculty development (not specific to international EM); flexibility in clinical schedules; and increased travel funds.”*

**Figure 1:**
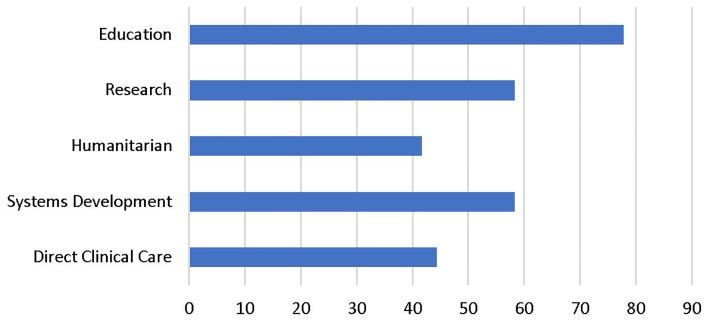
Project types during international emergency medicine fellowship.

Most respondents worked in EM education followed by health systems development and research with the fewest respondents involved in direct clinical care and humanitarian response during their fellowship fieldwork (Figure 1).

**Figure 2:**
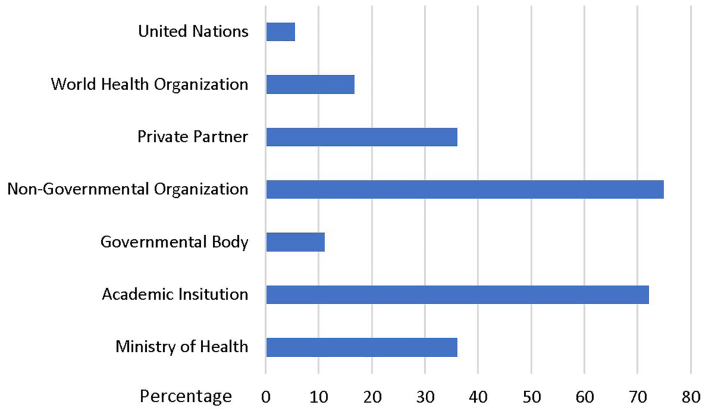
Type of fieldwork organizations with which the fellows worked.

More than half of respondents worked with either non-governmental organizations or academic institutions (Figure 2).

**Figure 3:**
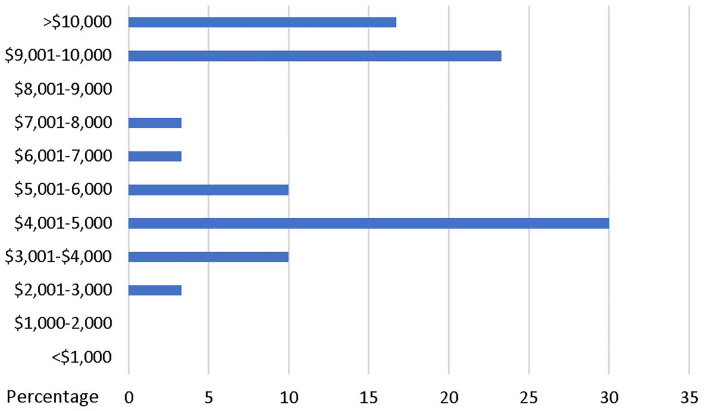
Amount of fellowship funding.^4^

During their fellowship, some level of funding was provided for 88.3% of the respondents for fieldwork. The amount of funding given to fellows is shown in Figure 3. Funding came from the fellow’s institution (75.0%), grants (16.7%), private partners (16.7%), and other sources (2.8%).

**Figure 4:**
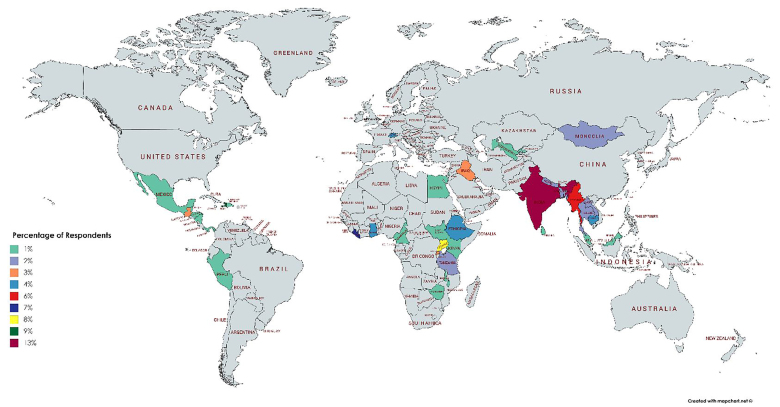
Geographic distribution of field sites (percentage per site 1–13%).

The geographic distribution of field sites is shown in the map below (Figure 4). The majority of respondents worked in India followed by sub-Saharan Africa with the least number of respondents working in the Americas.

Of those who responded, 80.6% reported they would complete the fellowship again. Overall the most valuable components of the fellowship were felt to be the advanced degree followed by developing contacts and networking. One respondent succinctly described the fellowship as an opportunity to *“form professional networks, greatly increase confidence as a researcher and gain experience teaching in LMICs*.” The biggest challenges faced during fellowship were the *“overwhelming burden of clinical duties which detracted from getting the most out of field opportunities and advanced degree,”* lack of IEM mentorship and no clear career path development, and lack of fieldwork opportunities.

### Post-Fellowship Results

The majority, 91.7%, of respondents, continue to work in global EM with 67.4% working in academics, 16.3% in community settings, and 11.6% in unspecified international settings. Respondents stated that the advanced degree they received during fellowship had provided skills to conduct research and obtain funding, further adding to their academic profile. Summation of the respondents’ use of their advanced degree was that “*[the degree] adds to my academic profile in my current department/faculty position, allows me to approach global health in a more comprehensive manner and to lend a public health perspective and approach to EM.”*

Those who have continued working in IEM work as part of the institutional division of international EM, IEM fellowship director, mentoring/teaching residents and medical students, international research, lecturing/planning international conferences, humanitarian work, education and training in international EM training programs, capacity development, and health system development. Those who did not continue international work cited lack of institutional support. Reasons for why they did not continue in IEM included the following: *“[F]inancial opportunity costs are too high given debt load*”; work-life balance; and limited academic positions domestically in international EM.

## DISCUSSION

Understanding why IEM fellows become fellows, where they do their fieldwork, their institutional experience, and postgraduate roles provide information for both fellowship directors and future fellows. These data can then be used for fellowship development and aligning future fellows’ expectations and goals with what is offered by the training programs. Our survey shows that most fellows choose a two-year fellowship and pursue an advanced degree, which many found to be the most valuable part of their fellowship. The data also suggest fellowship decision-makers should focus on providing opportunities and time to pursue advanced degrees with a focus on epidemiology and biostatics as many respondents felt that these were gaps in the master’s degree programs.

Balancing clinical hours and field time was a constant challenge for fellowship directors and significantly impacted the fellows’ experience. Acquiring protected fieldwork time for fellows is traditionally tied to the overall support of the home institution and requires active negotiations between fellowship and departmental leadership. The IEMF Consortium could play a more active role in developing advocacy tools for fellowship directors to assist in these negotiations.

Most fellowship activities took place in India and sub-Saharan Africa. Although it can be difficult to build global partnerships, the network of current and past fellows’ projects might be a resource to build future partnerships and networking opportunities in areas not currently linked to IEM programs.

Almost 20% of respondents reported that they would not complete the fellowship again mainly because of financial concerns. Financial concerns occurred both during the fellowship and post-fellowship periods. Both the monetary value of fieldwork and the opportunity costs of only receiving a fellowship salary out of residency were cited as key factors. Funding for fieldwork was seen as inadequate as the funding provided to fellows is used for travel expenses related to fieldwork but also to cover educational activities, such as conferences and potentially master-level courses, publication fees, and costs related to fieldwork projects. Fellowship directors and departmental leadership should consider these concerns when developing the fellow’s salary and procuring travel funds in order to keep the fellowship competitive. Post-fellowship, most fellows continued to pursue global health work; those who did not left IEM due to the high cost burden relative to the benefits of continuing international work. To help those who train in this new field continue as part of the IEM community post fellowship, mentorship and funding opportunities should be shared and developed.

## LIMITATIONS

The primary limitation of this study was the limited response rate. This may have skewed results to those who have continued to pursue global health. Another limitation was the potential for recall bias given that some fellows had graduated almost a decade prior. The sampling technique was limited by the completeness of fellowship directors’ responses. Additionally, not all fellowships were included in this study as only active fellowships within the IEMF Consortium were contacted. This may have resulted in missed respondents from inactive or former fellowships limiting the sample size. It was assumed that fellowship directors had emails to previous IEM fellows, but lack of email addresses by the fellowship directors could have also posed a problem in generating an accurate sampling of fellows.

## CONCLUSION

This study provides much needed information on the experience of international emergency medicine fellows and the international EM fellowship. IEM fellows traditionally have completed more two-year fellowships with a slight minority entering fellowship with a second language. These fellows spent 26–50% of their time in the field with 83.3% of institutions providing funding. Financial cost of continuing international work was cited as the main challenge in pursuing an IEM fellowship, which may be mitigated with novel approaches to funding global health work and improved departmental support. IEMFs should prioritize field preparation training, funded fieldwork, and integrated master-level qualifications to support the further development of this subspecialty.

## Figures and Tables

**Table 1:** Demographics of survey respondents regarding their experiences in an international emergency medicine fellowship.

Variable	N(%)
Gender	
Male	16(44.4)
Female	20(55.6)
Age	
25–34	14(38.9)
35–44	20(55.6)
45–54	2(5.6)
>55	0(0.0)
Degree before fellowship	
MD	34
MPH	9
PhD	0
MS	1
DO	1
Other (MBA, MA Bioethics)	2
Languages spoken before fellowship	
French	8(12.5)
Spanish	17(26.6)
Other (Haitian Creole, Mandarin, Kiswahili)	3(4.7)

*MD*, doctor of medicine; *MPH*, master of public health; *PhD*, doctor of philosophy; *MS*, master in science; *DO*, doctor of osteopathic medicine; *MBA*, master of business administration; *MA*, master of arts.

**Table 2:** Descriptive analysis of international emergency medicine fellowships.

Variable	N(%)
Length of fellowship	
1 year	11(30.6)
2 years	25(69.4)
Degree obtained during fellowship	
Yes	23(63.9)
No	13(36.1)
Degree earned during fellowship	
Master of public health	16(44.4)
Doctor of philosophy	0(0)
Master of science	3(8.3)
Master of academic medicine	1(2.8)
Diploma of tropical medicine	4(11.1)
Diploma in humanitarian assistance	1(2.8)
Faculty appointment during fellowship	
Yes	31(88.5)
No	4(11.1)
No answer	1
Percentage of fieldwork during fellowship	
0–25%	16(44.)
26–50%	18(50.0)
51–75%	2(5.6)
>75%	0
Allocated fieldwork funding	
Yes	30(83.3)
No	6(16.7)
Existing field sites	
Yes	32(88.9)
No	4(11.1)
Participation in existing field sites	
Yes	29(80.6)
No	3(8.3)
No answer	4 (11.1%)
Fieldwork deliverables	
Formal research	14(38.9)
Educational curricula	12(33.3)
Quality improvement / Quality assurance	6(16.7)
Field report	17(47.2)
Other	1(2.8)
None	9(25.0)

1 Existing field sites are sites that the fellow’s institution had an agreement with to place fellows for fieldwork.

2 Fellows who worked in institutions’ existing field sites vs creating a new field site or working with an organization outside the institution’s fieldwork sites.

3 Some fellowships had multiple deliverables; therefore, one respondent could have multiple deliverables.

4 Fellows received funding from a variety of sources used for educational pursuits such as master-level courses, conferences, publication fees, and travel associated with fieldwork.
